# Robot-assisted esophagectomy (RAE) versus conventional minimally invasive esophagectomy (MIE) for resectable esophageal squamous cell carcinoma: protocol for a multicenter prospective randomized controlled trial (RAMIE trial, robot-assisted minimally invasive Esophagectomy)

**DOI:** 10.1186/s12885-019-5799-6

**Published:** 2019-06-21

**Authors:** Yang Yang, Xiaobin Zhang, Bin Li, Zhigang Li, Yifeng Sun, Teng Mao, Rong Hua, Yu Yang, Xufeng Guo, Yi He, Hecheng Li, Hezhong Chen, Lijie Tan

**Affiliations:** 1 Department of Thoracic Surgery, Shanghai Chest Hospital, Shanghai Jiao Tong University, No. 241, Huaihai West Rd, Shanghai, 200030 China; 2 Department of Thoracic Surgery, Ruijin Hospital Affiliated to Shanghai Jiao Tong University School of Medicine, No. 197, Ruijin Er Rd, Shanghai, 200025 China; 3 Department of Thoracic Surgery, Changhai Hospital Affiliated to The Second Military Medical University, No. 168, Changhai Rd, Shanghai, 200433 China; 4 Department of Thoracic Surgery, Zhongshan Hospital Affiliated to Fudan University, No. 180, Fenglin Rd, Shanghai, 200032 China

**Keywords:** Robot-assisted surgery, Thoracoscopic esophagectomy, Efficacy, Complications, Quality of life

## Abstract

**Background:**

Currently, there are three main surgical approaches for resectable esophageal cancer: open transthoracic esophagectomy (OTE), conventional minimally invasive esophagectomy (MIE) and robot-assisted esophagectomy (RAE). Previous studies had demonstrated the better short-term outcomes in MIE or RAE when compared to OTE, respectively. However, to date, no prospective study was designed to compare these two minimally invasive approaches (MIE and RAE). The primary objective of this study is to compare the outcomes on survival, safety and efficacy, quality of life between RAE and MIE in the treatment for resectable esophageal squamous cell carcinoma (ESCC).

**Methods:**

This study is designed as a multicenter, prospective, randomized, non-inferiority phase III clinical trial, investigating the safety and efficacy of RAE compared with MIE in the treatment of resectable ESCC. Eligible patients are randomly assigned to either RAE (*n* = 180) or MIE (*n* = 180) group. The follow-up visits will be scheduled at 3, 6, 9, and 12 months in the first two years, and then every 6 months until the end of the study. During the follow-up period, clinical data and quality of life questionnaires will be examined. The primary endpoint is the 5-year overall survival (OS). The secondary endpoints are 3-year OS, 5-year disease-free survival (DFS), short-term outcomes as well as quality of life.

**Discussion:**

This is the first prospectively randomized controlled trial designed to compare RAE with MIE as surgical treatment for resectable ESCC. According to our hypothesis, RAE will result in at least similar oncologic outcomes and long-term quality of life, but with a shorter operation time, lower percentage of perioperative complications, lower blood loss, and shorter hospital stay when compared with MIE. This study started in July 2017. Follow-up will terminate after 5 years from the time when the last patient was enrolled.

**Trial registration:**

ClinicalTrial.gov: NCT03094351 (March 29, 2017). The trial was prospectively registered.

## Background

Esophageal cancer is the seventh common malignant tumor and ranks sixth in tumor-related mortality worldwide in 2018. In terms of the histological subtypes, adenocarcinoma is frequently observed in Europe and the United States, while squamous cell carcinoma is the predominant form of Asian populations [1]. Although great advances have been made in the diagnosis and treatment of esophageal cancer, the overall 5-year survival only ranges from 15 to 25% [2]. Radical esophagectomy and lymphadenectomy has been widely acknowledged with paramount importance in cure-intended therapy for esophageal squamous cell carcinoma (ESCC).

Currently, there are three surgical approaches to perform a radical esophagectomy: open transthoracic esophagectomy (OTE), conventional minimally invasive esophagectomy (MIE) and robot-assisted esophagectomy (RAE). MIE is now the preferred surgical approach worldwide, which has brought better postoperative outcome by its minimally invasive feature [3, 4]. Besides, reduced morbidity and mortality rates have been reported when compared with the conventional open approach by previous studies [5, 6]. Apart from observational studies, there are several prospective trials demonstrated the advantages of MIE [7–9]. However, several problems still have not been solved. First, short-term outcomes were not obviously improved in esophageal cancer patients undergoing MIE [10]. Second, the oncological results of MIE remain to be proven, as no RCTs have demonstrated the long-term survival between MIE and OTE [11]. Third, several population-based studies have reported a higher re-intervention rate in patients who underwent MIE, while no evident reduction in postoperative complications or overall morbidity was observed [9, 12].

In 2003, RAE was developed at the University Medical Center Utrecht (UMCU) to overcome the technical limitations of MIE [13]. Based on the advantages of enlarged and three-dimensional view, RAE could facilitate the complex procedures in conventional thoracoscopic approach [14]. With the progress of robot technology during the past decade, RAE has become more popular in the surgical treatment for esophageal cancer [15, 16]. In terms of treatment outcomes, previously observational studies have shown that RAE is at least equivalent to OTE [17]. Up to date, prospective trials to compare RAE with OTE in the treatment of esophageal cancer are infrequent. The ROBOT trial was a randomized trial which aims to evaluate the safety and efficacy of RAE as an alternative technique to OTE for surgical treatment of esophageal cancer. The results demonstrated that RAE resulted in a lower incidence of postoperative complications and better quality of life when compared to OTE. Moreover, the two surgical techniques have comparable and satisfactory oncological results [18]. However, whether RAE can improve the outcomes and surpass the conventional MIE remains to be demonstrated.

To the best of our knowledge, no randomized studies were conducted to compare RAE with MIE in patients with resectable ESCC (cT_1-4a_N_0-2_ M_0_) to date. Hence, we launch this multicentric phase III prospective clinical trial to assess the potential advantages of RAE versus MIE in the surgical treatment for patients with ESCC.

## Objective

This is a multicenter, prospective, randomized, open and parallel controlled, non-inferiority trial, which aims to compare RAE versus MIE in the treatment for patients with ESCC.

## Methods

### Study design

**Fig. 1:**
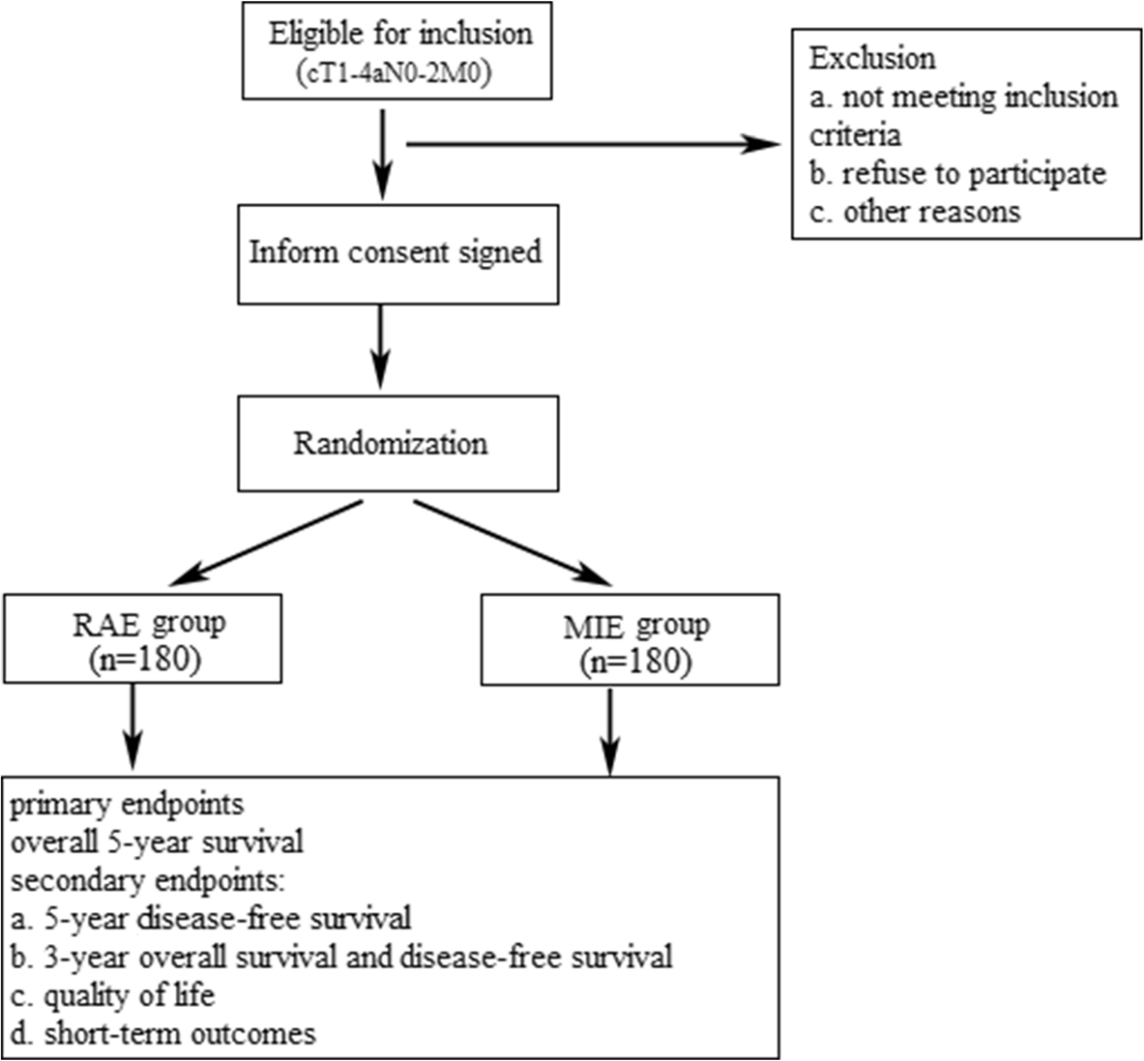
Flow chart of the RAMIE trial

This is a prospectively randomized controlled trial to assess the comparison between RAE and MIE in the treatment for patients with ESCC. According to previous studies, the long-term survival results of RAE seemed to be comparable but not inferior to the MIE or open esophagectomy [19, 20]. Therefore, the present study was designed as a non-inferiority trial, which was based on the hypothesis that the 5-year overall survival in the RAE is uncompromised with MIE. To achieve the primary goal, 360 patients with ESCC will be recruited from 4 high-volume centers (> 100 cases of combined approaches annually) in China. Based on the volume of esophagectomy in our center, 120 patients will be scheduled for this trial and approximately 5 patients will be enrolled for each group per month. For the other three centers, the estimated enrolled patients will be 2 for each group per month. The flow chart of this trial is presented in Fig. 1. The study has been approved by the ethics committees of participating institutions, as well as in accordance with the principles of the Declaration of Helsinki and Good Clinical Practice Guidelines. Written informed consent is obtained from each participant.

Neoadjuvant therapy will be used as a stratification factor in this study. After finishing preoperative treatment, patients will be restaged and confirmed to suitable for surgery by multidisciplinary team (MDT) of clinicians. When the tumor is considered as resectable, patients will undergo the randomized intervention - either RAE or MIE depending on randomization.

The study started on July 2017. Enrollment will take about 2 years and follow-up for will be 5 years. So the total duration of this study will lasts 7 years.

### Study population

Patients with thoracic ESCC will be considered as eligible for this trial. Investigators from each institution will take charge of the enrollment according to the inclusion/exclusion criteria and patients’ conditions.

### Inclusion criteria

1. Age ranges from 18 to 75 years;
2. European Clinical Oncology Group Performance Status (ECOG PS) 0–2;
3. Histological subtype of esophageal squamous cell carcinoma;
4. Primary tumors are located at the intrathoracic esophagus;
5. Pre-treatment stage as cT_1-4a_N_0-2_ M_0_ (AJCC/UICC 7th Edition);
6. Didn’t receive any therapy including chemotherapy or radiation for other cancers;
7. Written informed consent.

### Exclusion criteria

1. Cervical esophageal cancer and carcinoma of gastro-esophageal junction;
2. Patients with unresectable or metastatic esophageal cancer;
3. Histological subtype of esophageal non-squamous cell carcinoma;
4. History of previous thoracic surgery;
5. Patients with other malignant tumor (previous or current);
6. Participation in another clinical trial during this study.

### Sample size considerations

The sample size was calculated based on the primary outcome overall survival. A non-inferiority trial is planned in which the primary analysis will use the non-inferiority log-rank test. Generally, non-inferiority margins in local-advanced cancers were in a narrow range (1.18–1.33) according to the FDA’s estimate [21]. After extensive discussion, the researchers have decided that the upper bound on non-inferiority is 1.33. The total trial will take about 7 years which consists of 2-year recruitment period and 5-year follow-up period. We estimated a missing rate of 5% per year in each group. A hazard rate of 0.60, a power of 0.80 and a significance level of 0.05 will be used. Based on the assumptions, a sample size of 360 (2 × 180) participants was calculated using PASS 11 software (NCSS, LLC; Version 11.0.7).

### Randomization

Randomization will be performed via a computer-generated random numbers sequence and further stratified by whether the patient has undergone neoadjuvant treatment or not. Considering the unfeasibility in clinical practice, no blinding will be set for the patients and surgeons. Nevertheless, the independent study team is blinded to the allocation.

### Surgical technique

All patients underwent RAE or MIE (McKeown procedure) with two-field lymphadenectomy by the same experienced surgeons who had entirely completed the learning curve in each center. A surgeon who accomplished 40 cases of RAE or MIE annually is determined to be sufficiently skilled for our study. RAE is completed using Da Vinci surgical system (Intuitive Surgical, Inc., Sunnyvale, CA, USA) and MIE is carried out with a thoracoscopic-laparoscopic system.

**Fig. 2:**
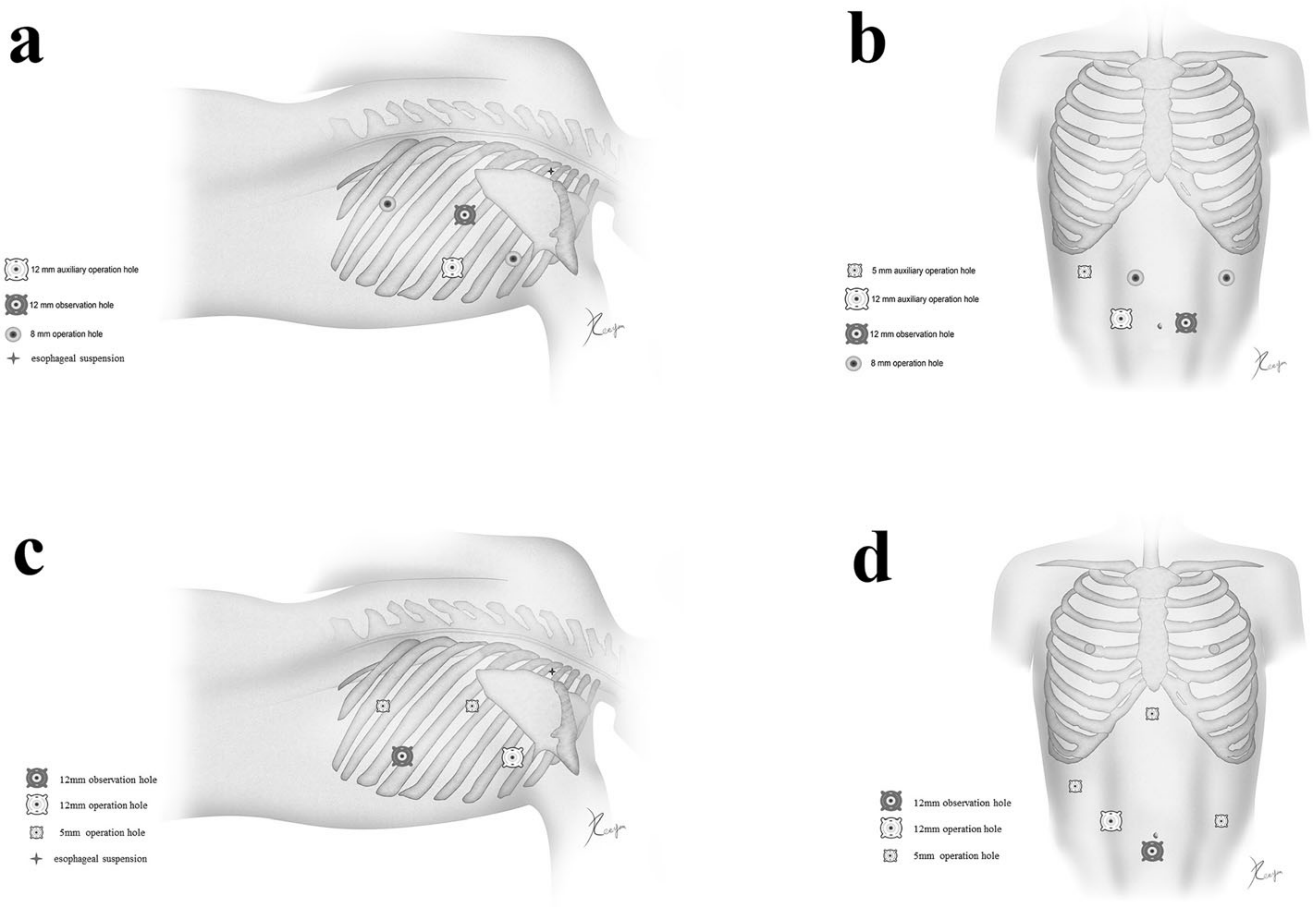
Trocars arrangement during the process of RAE and MIE. **a** thoracic part of RAE. **b** abdominal part of RAE. **c** thoracic part of MIE. **d** abdominal part of MIE. The images depicted in Fig. 2 derive from our own source

For RAE, the procedure in details has been described in our previous article [22]. For the thoracic phase, patients are placed in the left semi-prone position and trocars arrangement are presented in Fig. 2a. The mediastinal pleura above the arch of the azygos vein is divided firstly. Lymph nodes along the right-RLN and the superior esophagus are dissected en bloc. Then the dissection continued from the right main bronchus up to the plane between the esophagus and the trachea membrane. Lymphadenectomy is performed along the left-RLN from the thoracic outlet to the aortic-pulmonary window. The subcarinal lymph nodes will be dissected after this step, and the lower esophagus is dissected to the hiatus. For the abdominal phase, patients are placed in the reverse Trendelenburg position, and the robotic cart will be docked from the head side of the patient. Five ports are used in the abdominal stage, including one observation port, two robotic arm ports and two assistant ports (Fig. 2b). The greater curve is dissected firstly until the short gastric artery. The celiac area is dissected and the left gastric artery is cut off. Then the operation converts to the hepatogastric ligament, in which the right crus, left crus as well as fundus are dissected. A small incision is made at the sub-xiphoid and a narrow gastric tube (3–4 cm) will be made from the abdomen. Finally, the anastomosis is completed at the neck.

For MIE, the procedure in details has also been published in previous article [23]. Trocars for the thoracic phase are placed as followed: the 7th intercostal of the right anterior axillary line is set as the observation port, the 3th intercostal is set as the primary operation port, the 6th intercostal of the right midaxillary line is set as the secondary operation port, and the 9th intercostal of the right midaxillary line is set as the operation port for the assistant surgeon (Fig. 2c). Trocars arrangement for the abdominal phase is shown in Fig. 2d. As in the RAE, the MIE procedure consists of thoracoscopic mobilization of the esophagus, which was followed by laparoscopy with creation of the gastric tube and cervical esophagogastric anastomosis.

### Study endpoints

#### Primary endpoint

The primary endpoint is the 5-year overall survival (OS) time. OS is defined as the time from the date of surgery to the day of death or to the last follow-up [24].

#### Secondary endpoints

1. 5-year disease-free survival (DFS) time: DFS is defined as the time from the date of surgery to the day of tumor recurrence, tumor progression or patients’ death assessed up to 5 years [24].
2. 3-year OS and DFS.
3. Quality of life (QOL): QOL is assessed among patients by using the European Organization for Research and Treatment of Cancer Quality of Life Questionnaire C-30 (EORTC QLQ-C30) and EORTC QLQ-OES18 at randomization, and 3 month, 6 month, 9 month and yearly till 3 years after surgery respectively.
4. Short-term outcomes: Short-term outcome refers to operation related index during the perioperative period, which includes operative time, total blood loss, R0 resection rate, total and positive numbers of dissected lymph nodes, 30- and 90-day postoperative mortality, length of hospital stay and ICU stay, postoperative recovery, and the incidence of treatment-related complications. True operation time is defined as time from incision until finally closure (minutes), and in which the time of docking and undocking robot was removed. We also assess the total operative time in which docking and undocking time was included. Postoperative recovery is defined as the days of chest tube and other surgical drainages removed, and the days until first ambulation and oral intake. First ambulation was defined as when the patient could continuously walk for at least 10 m, and first oral intake was when the patient could first eat gelatin or semi-solid food. The complications were diagnosed and defined according to the International Consensus of Esophagectomy Complications Consensus Group (ECCG) [25] (shown in Table 1).

**Table 1:** Definitions of complications

Pulmonary	
Pneumonia: Radiographic confirmation with positive respiratory tract culture.	
Pleural effusion: Pleural effusion requiring additional drainage procedure.	
Pneumothorax: Radiographic confirmation requiring chest tube reinsertion.	
Respiratory failure: Reintubation or tracheostomy for weaning failure.	
Atelectasis: Atelectasis mucous plugging requiring bronchoscopy.	
Cardiovascular	
Cardiac arrest requiring CPR	
Atrial arrhythmia: Electrocardiographic (ECG) confirmation of atrial arrhythmia.	
Deep venous thrombosis: Ultrasound confirmation of deep venous thrombosis.	
Myocardial infarction: Confirmed by ECG changes, information with cardiac biomarkers and symptoms of ischaemia.	
Gastrointestinal	
Anastomotic leak: Full thickness defect involving esophagus, anastomosis, staple line, or conduit. Detection of saliva, ingested material, gastric secretions, or bile in the drain or wound.	
Conduit necrosis: Identified endoscopically. Extensive conduit necrosis has to be treated by conduit resection with diversion.	
Diaphragmatic hernia: Radiography confirm the presence of abdominal organs in the thoracic cavity, with or without gastrointestinal symptoms.	
Vocal cord palsy: Any sign of voice changes or aspiration. Confirmation and assessment should be by direct examination, sometimes laryngoscopy is necessary. Severe injury requiring acute surgical intervention (due to aspiration or respiratory issues).	
Chyle leak: Chyle test is positive in the thoracic drainage. Treated with enteric dietary modifications, total parenteral nutrition, and nterventional or surgical therapy.	
Wound infection: Local findings of erythema, drainage, subcutaneous emphysema, or tenderness requiring wound opening or antibiotics.	
Delirium: Transient confusion confirmed by disturbances in consciousness, cognition, and perception.	

### Assessment and follow-up

Pre-treatment assessment is performed by routine examinations including a physical examination, standard laboratory tests, a CT scan of the thorax and abdomen (or PET/CT), upper gastrointestinal contrast, upper endoscopy with biopsies and endoscopic ultrasound. For patients who had received preoperative neoadjuvant treatment, they will be restaged and confirmed to suitable for surgery by clinicians.

After surgery, follow-ups will be performed one time per 3 months in the first two years and one time per 6 months from the third year until the end of follow-up. Examination will be focused on laboratory tests (blood routine, tumor biomarker), CT scan, ultrasound, and quality of life questionnaires. For all patients, follow-up is carried out until the end of the trial or death.

### Statistical analysis

Statistical analyses are performed using SPSS version 20.0 software (SPSS Inc. Chicago, Illinois, USA). Survival will be estimated by Kaplan-Meier methods and analyzed using log-rank test. Complications will be calculated as percentage with 95% confidence intervals and analyzed by χ^2^ test or Fisher’s exact test. Student’s *t* test and analysis of variance (ANOVA) test was conducted for the comparison in continuous variables, while χ^2^ test or Fisher’s exact test was used in categorical variables. *P* < 0.05 is set as the significance level.

### Current status

The RAMIE trial has been ethically approved by the ethics committees of Shanghai Chest Hospital (KS1734). Recruitment of patients was started in July 1st, 2017. It is still at the stage of recruiting as 210 patients have been recruited until December 1st, 2018.

## Discussion

Minimally invasive esophagectomy, including MIE and RAE, have been used to reduce postoperative complication and improve postoperative recovery compared with open esophagectomy. Theoretically, RAE seems to get better short-term and oncological results, which could be supported by the facilitated manipulation and precise dissection of robotic system [26]. However, controversy still exists on the potential advantages of RAE versus MIE. To the best of our knowledge, this is the first randomized controlled trial designed to compare RAE with MIE as surgical treatment for patients with resectable ESCC.

Surgical resection with radical lymphadenectomy remains a critical element in the treatment of esophageal cancer. Therefore, the majority of studies concerning the application of RAE and MIE have focused on the extent of lymphadenectomy, especially for mediastinal lymph node dissection. Several retrospective studies demonstrated that RAE yielded significantly more lymph nodes than MIE especially in the upper mediastinum [19, 27, 28]. Lymph nodes dissection along the bilateral recurrent laryngeal nerve (RLN) is key step due to its high metastatic rate, but it is technically challenging because of narrow operative space. The feasibility and safety of the robot-assisted lymphadenectomy along the bilateral RLNs were demonstrated in a previous study [29]. Chao et al. also showed that compared with MIE, RAE resulted in a higher lymph node yield along the left RLN without increasing morbidity [28]. Thoracoscopic instruments have their limitations with respect to dissection in a small and narrow space. Instead, robotic surgery has several technological advantages as it provides a three-dimensional view, ten times magnification, tremor control, and ambidexterity.

The advantage of robotic system gives surgeons greater confidence of achieving a complete bilateral RLN lymph nodes dissection, but this may increase the incidence of postoperative RLN injury. Studies show the postoperative RLN injury rate range from 9.1 to 20.6% in RAE group as from 3.8 to 29.4% in MIE group, there is no significant difference between the two groups [19, 27, 28, 30]. The left RLN injury is more common than the right RLN. Notably, RLN injury can be caused by contusions, excessive stretching, and thermal damage occurring during manipulation. Chao et al. reported their method reducing the RLN injury by using the third robot arm (controlled by the operator) which allows achieving an excellent operating exposure through the application of stable and self-controllable tractions and countertractions on the esophagus and trachea [28].

Since robotic surgery has just begun to be accepted by thoracic surgeon, several groups show that the operation time of robot-assist esophagectomy is significantly higher than that of thoracoscopic esophagectomy, especially one-lung ventilation time [19, 30]. This has the potential to increase postoperative respiratory complications, but no significant increase in the risk of respiratory complications was identified in the RAE group. These studies also have found that no significant increase in the risk of anastomotic leakage, or other postoperative complication. For the reason that the surgeon can perform complex procedures sitting comfortably, they have not felt fatigued even if the prolong of operative time.

Although RAE is reported to improve the efficiency of lymph node dissection, it is unclear whether more extensive lymph node dissection could result in a significant survival advantage. Fewer studies were designed to focus on comparing long-term survival between RAE and MIE. Park et al. reported that the 5-year overall survivals were not different between the two groups (69% in RAE vs. 59% in MIE, *P* = 0.737). The 5-year freedom from locoregional recurrence was 88% in the RAE group and 74% in the MIE group, however the difference was not statistically significant (*P* = 0.100, 19]. The primary endpoint of our controlled trial is 5-year overall survival. According to our hypothesis, RAE will result in at least similar oncological outcomes and long-term quality of life compared with MIE.

## Data Availability

This article has used no dataset. Therefore, no additional data files are available.

## Abbreviations

AJCC: American Joint Committee on Cancer
CT: Computed tomography
DFS: Disease-free survival
ECCG: Esophagectomy Complications Consensus Group
ECOG: European Clinical Oncology Group
EORTC: European Organization for Research and Treatment of Cancer
ESCC: Esophageal squamous cell carcinoma
MDT: Multidisciplinary team
OS: Overall survival
OTE: Open transthoracic esophagectomy
PET-CT: positron emission tomography-computed tomography
QOL: Quality of life
RAE: Robot-assisted esophagectomy
RAMIE: Robot-Assisted Minimally Invasive Esophagectomy
RLN: Recurrent laryngeal nerve
UICC: International Union for Cancer Control

## References

[1] Fitzmaurice C, Allen C, Barber RM, Barregard L, Bhutta ZA, Brenner H, Dicker DJ, Chimed-Orchir O, Dandona R, Global Burden of Disease Cancer C (2017). Global, regional, and National Cancer Incidence, mortality, years of life lost, years lived with disability, and disability-adjusted life-years for 32 Cancer groups, 1990 to 2015: a systematic analysis for the global burden of disease study. JAMA Oncol.

[2] Pennathur A, Gibson MK, Jobe BA, Luketich JD (2013). Oesophageal carcinoma. Lancet.

[3] Safranek PM, Cubitt J, Booth MI, Dehn TC (2010). Review of open and minimal access approaches to oesophagectomy for cancer. Br J Surg.

[4] Straatman J, van der Wielen N, Cuesta MA, Daams F, Roig Garcia J, Bonavina L, Rosman C, van Berge Henegouwen MI, Gisbertz SS, van der Peet DL (2017). Minimally invasive versus open esophageal resection: three-year follow-up of the previously reported randomized controlled trial: the TIME trial. Ann Surg.

[5] Nafteux P, Moons J, Coosemans W, Decaluwe H, Decker G, De Leyn P, Van Raemdonck D, Lerut T (2011). Minimally invasive oesophagectomy: a valuable alternative to open oesophagectomy for the treatment of early oesophageal and gastro-oesophageal junction carcinoma. Eur J Cardiothorac Surg.

[6] Sihag S, Wright CD, Wain JC, Gaissert HA, Lanuti M, Allan JS, Mathisen DJ, Morse CR (2012). Comparison of perioperative outcomes following open versus minimally invasive Ivor Lewis oesophagectomy at a single, high-volume Centre. Eur J Cardiothorac Surg.

[7] Biere SS, van Berge Henegouwen MI, Maas KW, Bonavina L, Rosman C, Garcia JR, Gisbertz SS, Klinkenbijl JH, Hollmann MW, de Lange ES (2012). Minimally invasive versus open oesophagectomy for patients with oesophageal cancer: a multicentre, open-label, randomised controlled trial. Lancet.

[8] Briez N, Piessen G, Bonnetain F, Brigand C, Carrere N, Collet D, Doddoli C, Flamein R, Mabrut JY, Meunier B (2011). Open versus laparoscopically-assisted oesophagectomy for cancer: a multicentre randomised controlled phase III trial - the MIRO trial. BMC Cancer.

[9] Seesing MFJ, Gisbertz SS, Goense L, van Hillegersberg R, Kroon HM, Lagarde SM, Ruurda JP, Slaman AE, van Berge Henegouwen MI, Wijnhoven BPL (2017). A propensity score matched analysis of open versus minimally invasive transthoracic Esophagectomy in the Netherlands. Ann Surg.

[10] Booka E, Takeuchi H, Kikuchi H, Hiramatsu Y, Kamiya K, Kawakubo H, Kitagawa Y (2019). Recent advances in thoracoscopic esophagectomy for esophageal cancer. Asian J Endosc Surg.

[11] Takeuchi H, Kawakubo H, Kitagawa Y (2013). Current status of minimally invasive esophagectomy for patients with esophageal cancer. Gen Thorac Cardiovasc Surg.

[12] Sihag S, Kosinski AS, Gaissert HA, Wright CD, Schipper PH (2016). Minimally invasive versus open Esophagectomy for esophageal Cancer: a comparison of early surgical outcomes from the Society of Thoracic Surgeons National Database. Ann Thorac Surg.

[13] van Hillegersberg R, Boone J, Draaisma WA, Broeders IA, Giezeman MJ, Borel Rinkes IH (2006). First experience with robot-assisted thoracoscopic esophagolymphadenectomy for esophageal cancer. Surg Endosc.

[14] Boone J, Schipper ME, Moojen WA, Borel Rinkes IH, Cromheecke GJ, van Hillegersberg R (2009). Robot-assisted thoracoscopic oesophagectomy for cancer. Br J Surg.

[15] Dunn DH, Johnson EM, Morphew JA, Dilworth HP, Krueger JL, Banerji N (2013). Robot-assisted transhiatal esophagectomy: a 3-year single-center experience. Dis Esophagus.

[16] Puntambekar S, Kenawadekar R, Kumar S, Joshi S, Agarwal G, Reddy S, Mallik J (2015). Robotic transthoracic esophagectomy. BMC Surg.

[17] van der Sluis PC, Ruurda JP, Verhage RJ, van der Horst S, Haverkamp L, Siersema PD, Borel Rinkes IH, Ten Kate FJ, van Hillegersberg R (2015). Oncologic long-term results of robot-assisted minimally invasive Thoraco-laparoscopic Esophagectomy with two-field lymphadenectomy for esophageal Cancer. Ann Surg Oncol.

[18] van der Sluis PC, Ruurda JP, van der Horst S, Verhage RJ, Besselink MG, Prins MJ, Haverkamp L, Schippers C, Rinkes IH, Joore HC (2012). Robot-assisted minimally invasive thoraco-laparoscopic esophagectomy versus open transthoracic esophagectomy for resectable esophageal cancer, a randomized controlled trial (ROBOT trial). Trials.

[19] Park S, Hwang Y, Lee HJ, Park IK, Kim YT, Kang CH (2016). Comparison of robot-assisted esophagectomy and thoracoscopic esophagectomy in esophageal squamous cell carcinoma. J Thorac Dis.

[20] van der Sluis PC, van der Horst S, May AM, Schippers C, Brosens LAA, Joore HCA, Kroese CC, Haj Mohammad N, Mook S, Vleggaar FP (2019). Robot-assisted minimally invasive Thoracolaparoscopic Esophagectomy versus open transthoracic Esophagectomy for Resectable esophageal Cancer: a randomized controlled trial. Ann Surg.

[21] Tanaka S, Kinjo Y, Kataoka Y, Yoshimura K, Teramukai S (2012). Statistical issues and recommendations for noninferiority trials in oncology: a systematic review. Clin Cancer Res.

[22] Zhang X, Su Y, Yang Y, Sun Y, Ye B, Guo X, Mao T, Hua R, Li Z (2018). Robot assisted esophagectomy for esophageal squamous cell carcinoma. J Thorac Dis.

[23] Li B, Yang Y, Sun Y, Hua R, Zhang X, Guo X, Gu H, Ye B, Li Z, Mao T (2018). Minimally invasive esophagectomy for esophageal squamous cell carcinoma-Shanghai chest hospital experience. J Thorac Dis.

[24] Tang H, Tan L, Shen Y, Wang H, Lin M, Feng M, Xu S, Guo W, Qian C, Liu T (2017). CMISG1701: a multicenter prospective randomized phase III clinical trial comparing neoadjuvant chemoradiotherapy to neoadjuvant chemotherapy followed by minimally invasive esophagectomy in patients with locally advanced resectable esophageal squamous cell carcinoma (cT3-4aN0-1M0) (NCT03001596). BMC Cancer.

[25] Low DE, Alderson D, Cecconello I, Chang AC, Darling GE, D'Journo XB, Griffin SM, Holscher AH, Hofstetter WL, Jobe BA (2015). International consensus on standardization of data collection for complications associated with Esophagectomy: Esophagectomy complications consensus group (ECCG). Ann Surg.

[26] Ruurda JP, van der Sluis PC, van der Horst S, van Hilllegersberg R (2015). Robot-assisted minimally invasive esophagectomy for esophageal cancer: a systematic review. J Surg Oncol.

[27] Deng HY, Huang WX, Li G, Li SX, Luo J, Alai G, Wang Y, Liu LX, Lin YD. Comparison of short-term outcomes between robot-assisted minimally invasive esophagectomy and video-assisted minimally invasive esophagectomy in treating middle thoracic esophageal cancer. Dis Esophagus. 2018;31(8). 10.1093/dote/doy012 29538633

[28] Chao YK, Hsieh MJ, Liu YH, Liu HP (2018). Lymph node evaluation in robot-assisted versus video-assisted Thoracoscopic Esophagectomy for esophageal squamous cell carcinoma: a propensity-matched analysis. World J Surg.

[29] Kim DJ, Park SY, Lee S, Kim HI, Hyung WJ (2014). Feasibility of a robot-assisted thoracoscopic lymphadenectomy along the recurrent laryngeal nerves in radical esophagectomy for esophageal squamous carcinoma. Surg Endosc.

[30] He H, Wu Q, Wang Z, Zhang Y, Chen N, Fu J, Zhang G (2018). Short-term outcomes of robot-assisted minimally invasive esophagectomy for esophageal cancer: a propensity score matched analysis. J Cardiothorac Surg.

